# RNP interfaces as regulatory “active sites”: a repurposing strategy for small molecules targeting viral 5′-untranslated regions

**DOI:** 10.3389/fmolb.2026.1773385

**Published:** 2026-05-26

**Authors:** Louis G. Smith, Solomon Attionu, Sudeshi M. Abedeera, Barrington Henry, Srinivasa Penumutchu, Blanton S. Tolbert

**Affiliations:** 1 Stellar-Chance Laboratories, Department of Biochemistry and Biophysics, Institute for RNA Innovation, University of Pennsylvania, Philadelphia, PA, United States; 2 Howard Hughes Medical Institute, Chevy Chase, MD, United States

**Keywords:** 5′-UTR, Alphafold 3, IRES, molecular dynamics, repurposing, RNA-protein, RNA-small-molecule, virtual screen

## Abstract

RNA-protein (RNP) complexes regulate nearly every stage of gene expression and play central roles in viral infection and human disease. Despite decades of research, relatively few small molecules (SMs) have been shown to modulate RNP assemblies with mechanistic understanding. A major challenge in targeting RNA arises from the absence of well-defined SM binding pockets analogous to the catalytic active sites that guide conventional protein-directed drug discovery. However, in many biological contexts, RNP interfaces function as the effective “active sites” of regulatory RNA, where RNA structure and protein recognition surfaces converge to regulate gene expression. In this Perspective, we argue that the major obstacle to therapeutic progress is the difficulty of identifying functionally and structurally characterized RNP interfaces within highly dynamic regulatory networks. To address this challenge, we propose an integrative discovery framework centered on RNP interfaces that integrates state-of-the-art structure prediction, molecular dynamics simulations, ensemble-based virtual screening, and orthogonal biophysical validation to enable rational repurposing of FDA approved SMs. Viral $5^{\prime }$ -UTR untranslated regions ( $5^{\prime }$ -UTRs) provide a compelling context for this strategy, as they function as structural scaffolds that present conserved RNP interfaces essential for translation and replication. By focusing on minimal RNP fragments that consist of recurrent structural motifs such as bulge loops, ensemble sampling can reveal transient pockets suitable for SM virtual docking. Using Enterovirus A-71 $5^{\prime }$ -UTR as an illustrative example, we outline how interface-guided modeling can prioritize SMs capable of modulating specific RNP interactions. This ensemble-guided framework offers a generalizable strategy for accelerating the development of RNP-targeted therapies in viral and disease contexts.

## 1 Introduction

Targeting RNA with small molecules (SMs) requires new conceptual and technological innovations to accelerate the discovery of modalities that both illuminate biological mechanisms and hold therapeutic promise. A central challenge in targeting RNA with SMs arises from the absence of well-defined ligand binding pockets analogous to catalytic sites that guide conventional protein-directed drug discovery (Schneekloth and Pettersson, 2024; Zafferani and Hargrove, 2021; Childs-Disney et al., 2022). Instead, many regulatory RNAs function through dynamic structural elements that lack stereochemically discrete binding cavities, making it difficult to predict the functional consequences of RNA-targeted SMs *a priori*. However, RNA rarely acts alone in the cellular environment. In many biological contexts, RNA exerts its regulatory function through RNA-protein (RNP) assemblies that orchestrate essential steps in gene expression under both normal physiological and pathological conditions.

RNP interfaces often function as the effective “active sites” of regulatory RNAs. Within RNP complexes, the interfaces can generate structurally and functionally constrained interaction surfaces that guide regulatory outcomes and provide potential footholds for SM intervention. Viewing these interfaces as functional active sites provides a conceptual framework for applying principles of structure-guided drug discovery to RNA-centered regulatory systems. Yet despite decades of research and substantial investment, the repertoire of SM modulators of RNA and RNPs remains limited (Schneekloth and Pettersson, 2024; Zafferani and Hargrove, 2021; Childs-Disney et al., 2022). This disparity in part reflects the persistent challenges in identifying RNP interfaces that are functionally relevant, structurally characterized, and amenable to SM intervention. Identifying such interfaces across complex regulatory landscapes therefore represents a critical step toward enabling structure-guided discovery of RNA-targeted SMs.

Addressing this challenge requires complementary strategies that build on sequencing-based approaches for mapping RNA structure, RNA-binding protein (RBP) interactions, and SM binding sites across the transcriptome. More than half of human transcripts are estimated to exhibit measurable RBP occupancy *in vivo*, as demonstrated by transcriptome-wide CLIP and RNA interactome capture studies (Van Nostrand et al., 2020). For viruses, the prevalence of RBP-RNA interactions is predicted to be even higher, as both viral and cellular proteins engage viral RNA genomes at nearly every stage of the replication cycle (Lee et al., 2021; Zhou et al., 2022). This pervasive and dynamic binding landscape complicates the identification of discrete, druggable RNP interfaces, particularly when interactions are transient, often multivalent, and embedded within larger regulatory networks.

Overcoming these challenges requires shifting RNP modulator discovery efforts away from screening high-resolution structures of intact assemblies and toward minimal structural fragments that faithfully recapitulate local RNP binding interfaces. Recent advances–exemplified by AlphaFold3 (AF3) – enable modeling of RNP complexes within broader RNA tertiary architectures, yielding predicted binding interfaces that incorporate stereochemical features imposed by native RNA context. These contextual features are often absent from experimental structures of short RNA fragments that focus primarily on sequence-specific recognition (Abramson et al., 2024). When integrated with Molecular Dynamics (MD) simulations and biophysical validation, such models provide a robust foundation for capturing the conformational ensembles of RNP interfaces and for guiding structure-based discovery for virtual screening. Together, this hybrid framework links predictive modeling with empirical observation, yielding an experimentally anchored view of RNP structure that captures the transient and allosteric behaviors most relevant to function.

**FIGURE 1:**
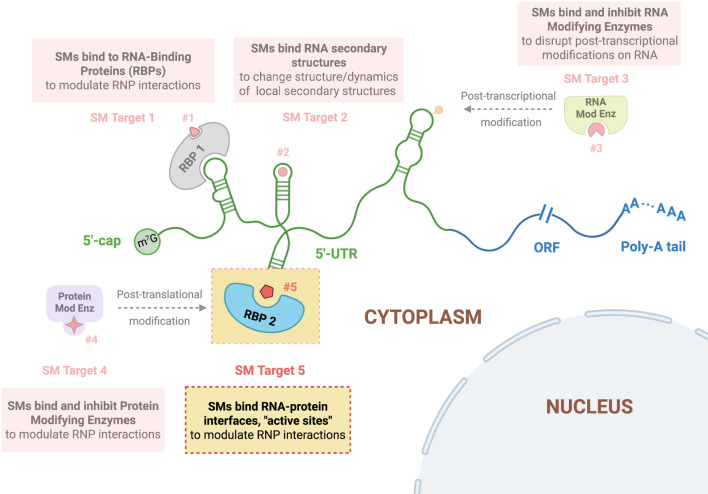
The viral $5^{\prime }$ -UTR constitutes an attack surface enriched in structured RNA elements that recruit RBPs to control essential steps of the viral life cycle. Within these assemblies, the RNP interfaces can function as regulatory “active sites” in which RNA structure and protein recognition surfaces converge to regulate viral gene expression. SMs that perturb these interfaces therefore have the potential to modulate viral replication. This Perspective focuses primarily on SMs that bind discrete RNA secondary structures within the viral $5^{\prime }$ -UTR and thereby modulate protein-RNA interactions (SM Target Class 5). However, additional mechanisms may also produce antiviral activities from repurposed SMs. These include SMs that bind RBPs at allosteric sites (SM Target Class 1), directly bind to isolated RNA structural elements (SM Target Class 2), perturb enzymes that modify viral $5^{\prime }$ -UTR (SM Target Class 3), or alter the activity of enzymes that regulate RBP function (SM Target Class 4). Collectively, these mechanisms highlight the various strategies by which SMs may modulate viral $5^{\prime }$ -UTRs.

Regulatory untranslated regions (UTRs), particularly $5^{\prime }$ -UTRs, provide a compelling biological context in which these regulatory RNP “active sites” are concentrated and functionally accessible (Figure 1). $5^{\prime }$ -UTRs integrate conserved secondary and tertiary structural motifs, chemical modifications, and dynamic protein interactions to control ribosome recruitment, translation efficiency, and cellular responses to stress and signaling cues (Figure 1). Dysregulation of $5^{\prime }$ -UTR-mediated control is implicated in cancer, neurodegeneration, and viral pathogenesis, underscoring their biological and therapeutic relevance (Castello et al., 2012).

Among both cellular and viral RNAs, $5^{\prime }$ -UTRs are enriched in structural elements such as stem loops, junctions, internal loops, and bulges that serve as docking platforms for RBPs and other regulatory factors. These features position $5^{\prime }$ -UTRs as focal points for identifying discrete, mechanistically interpretable RNP interfaces within otherwise complex transcript-wide interaction networks. Critically, the abundance of viral mRNAs increases as replication proceeds, rendering their $5^{\prime }$ -UTRs effective binding partners for modest affinity ligands through mass action effects (Boersma et al., 2020; Kamel et al., 2021; Castello and Iselin, 2023; Lee et al., 2025). This mass-action effect is particularly advantageous for drug repurposing, as it allows compounds with sub-micromolar affinities—often found in FDA-approved libraries—to exert antiviral effects when the targeted RNA is at high concentrations due to infection.

Within these regulatory regions, bulge loops emerge as recurrent and functionally significant RNA structural motifs. Local deviations from canonical helices expose unpaired nucleotides that frequently serve as recognition elements for RBPs and other ligands (Hermann and Patel, 2000; Dibrov et al., 2012; Davila-Calderon et al., 2020). The conformational plasticity of bulge loops enables diverse binding modes, allowing them to accommodate proteins, SMs, and other RNA elements with distinct stereochemical features. This also makes them good options for repurposing campaigns, because many binding-sites are available that may have an impact if a moderate affinity ligand is found. Surveys of experimentally determined RNA structures indicate that bulges and internal loops together constitute a substantial fraction (about 20%–30%) of local non-helical structural motifs. Large-scale annotation efforts based on curated RNA structures, including bpRNA, consistently identify bulges and internal loops as among the most prevalent non-Watson Crick elements across structured RNAs (Danaee et al., 2018). In viral genomes, where compact coding constraints favor conserved multifunctional RNA elements, bulges are particularly enriched within UTRs and internal ribosome entry site (IRES), where they function as adaptable platforms for host and viral factor recruitment (Leppek et al., 2018).

Consistent with this structural prevalence, curated RNA-ligand datasets reveal that bioactive SMs disproportionately engage RNAs containing bulges and internal loops rather than fully base-paired helices (Donlic et al., 2022). These motifs present unique stereochemical environments characterized by exposed bases, backbone flexibility, and transient pocket formation, making them attractive targets for SM recognition and RNP modulation (Hermann, 2016).

Here, we advance a conceptual framework integrating structure prediction, molecular simulation, and experimental validation to find binders among the FDA approved SMs. We discuss this framework using the $5^{\prime }$ -UTR of Enterovirus A-71 (EV-A71) as a representative example. Our proposed workflow integrates structural and computational methodologies to pinpoint mechanistic vulnerabilities in viral RNA regulation and accelerate the rational repurposing of clinically relevant compounds for antiviral intervention.

## 2 Viral $5^{\prime }$ -UTRs are an untapped attack surface for repurposed drugs

The $5^{\prime }$ -UTRs of viral RNAs play crucial roles at multiple stages of the viral life cycle, including the regulation of translation, genome replication, and RNA packaging (Dubois et al., 2018). This is particularly evident in positive-sense single-stranded RNA viruses, where the viral genome itself serves as mRNA (Leppek et al., 2018). The secondary structures of many viral $5^{\prime }$ -UTRs are highly conserved and fold into multiple stem-loops (SLs) and comprise essential regulatory elements, such as internal ribosome entry site (IRES) for cap-independent translation, signals that control genome replication, and specific motifs recognized by viral proteins during virion assembly (Martinez-Salas et al., 2018). Both structured and unstructured regions within viral $5^{\prime }$ -UTRs harbor binding sites for cellular RBPs and microRNAs that modulate viral gene expression and replication. Because viral $5^{\prime }$ -UTRs recruit and repurpose host RBPs to compensate for compact genome sizes (2–30 kb) and limited coding capabilities, they represent attractive targets for antiviral strategies aimed at disrupting essential host RBP-viral RNA interactions (Hanson et al., 2024).

### 2.1 EV-A71

A well-characterized example of a viral $5^{\prime }$ -UTR functioning as a regulatory hub is found in Enterovirus A-71 (EV-A71), where the $5^{\prime }$ -UTR coordinate both genome replication and translation. This region is comprised of a cloverleaf structure (SLI) that regulates viral RNA replication and the IRES (SLII-VI) that directs cap-independent translation. Within the IRES, cellular proteins, commonly referred to as IRES trans-acting factors (ITAFs), bind discrete structural elements to positively or negatively modulate translation, while a distinct set of host proteins interact with SLI to control replication. To date, more than 20 cellular RBPs have been identified that interact with the EV-A71 $5^{\prime }$ -UTR and collectively regulate viral replication and gene expression (Davila-Calderon et al., 2024).

Among the IRES elements, SLII is the most extensively characterized at the molecular level and remains the only region for which specific binding sites of multiple ITAFs have been mapped in detail. Notably, hnRNP A1 and AUF1 bind competitively to a bulge loop within SLII, enhancing and suppressing IRES-mediated translation, respectively. The same bulge has been successfully targeted by the small molecule antiviral DMA-135, which binds the RNA and stabilizes the host AUF1-SLII interaction, thereby shifting the SLII regulatory axis towards translation repression and reduced viral replication (Davila-Calderon et al., 2020). Collectively, these observations illustrate how selective perturbation of host RBP-viral $5^{\prime }$ -UTR interactions by SMs can potentially yield therapeutically meaningful outcomes.

The regulatory architecture of the EV-A71 $5^{\prime }$ -UTR is reflected in other medically important RNA viruses. In HIV-1 and SARS-CoV-2 the $5^{\prime }$ -UTRs contain conserved structural elements that coordinate translation, replication, and genome packaging through interactions with viral and host RBPs While no FDA-approved SMs currently target viral RNAs in these prevalent infections, several compounds have emerged as promising lead candidates capable of engaging unique architectural folds within their $5^{\prime }$ -UTRs (Giorgio and Duca, 2019; Zafferani et al., 2021; Sztuba-Solinska et al., 2014). Taken together, these systems reinforce that viral $5^{\prime }$ -UTRs recurrently present functionally conserved RNP interfaces that can, in principle, be prioritized as targets in a SM repurposing strategy as outlined here for EV-A71.

## 3 There is evidence that FDA approved molecules bind to RNAs and modulate RNA-protein complexes

Although developing SMs that bind RNA has been a challenge for conventional pipelines, there are numerous examples of SMs of both endogenous and exogenous origin that have been found to bind RNPs in cells. Endogenous small molecules such as adenosine triphosphate (ATP), guanosine triphosphate (GTP), and S-adenosylmethionine (SAM) regulate various cellular processes such as RNA localization, translation, and mRNA processing via mediation of RBP-RNA interactions (Miao et al., 2025). There are several examples of engineered RNA binding SMs, as well. In 2020, the designed splice modulator risdiplam was approved by the Food and Drug Administration (FDA); it is the only non-ribosomal RNA-targeting FDA approved small molecule to date (Mullard, 2020; Ratni et al., 2018; 2021). Risdiplam stabilizes the interaction between U1 small nuclear ribonucleoprotein and survival motor neuron (SMN) mRNA, leading to replenished SMN2 protein levels for proper maintenance of motor neurons (Ratni et al., 2018; 2021). Similarly, the amiloride DMA-135 stabilizes the RNP interaction between AUF1 and stem loop 2 of EV-A71 internal ribosome entry site (IRES), thereby repressing cap-independent translation of important viral proteins (Davila-Calderon et al., 2020).

The dearth of RNA-targeting FDA-approved small molecules can partly be ascribed to the conformational heterogeneity of RNAs, as well as the relatively fewer RNA structures deposited in the Protein Data Bank (approximately 26 protein structures for every single RNA structure). Encouragingly, recent RNA-SM screening campaigns have identified some protein inhibitors that bind RNAs as well. In one such case, Velagapudi et al. (2018) found that mitoxantrone, a topoisomerase II inhibitor, binds to the oncogenic pre-miR-21 RNA. The selective estrogen receptor modulator (SERM) raloxifene has also been shown to bind the $5^{\prime }$ -UTR of the hHBV ϵ RNA (LeBlanc et al., 2022). In fact, it was previously shown that several protein-binding small molecules possess significant chemical structure similarities to RNA-binders, and that these proteins do bind RNA in the cell, hinting at mechanisms by which these SMs may cause side-effects (Fang et al., 2023).

Levofloxacin is a notable example from that study. It is an FDA-approved oral antibiotic that functions as an inhibitor of the bacterial enzymes, DNA gyrase and topoisomerase IV (Croom and Goa, 2003). A few common side effects associated with the consumption of this SM include: nausea, neuropathy, and difficulty breathing. Fang et al. (2023) demonstrated that Levofloxacin has pervasive interactions throughout the transcriptome. In addition, they demonstrated that RNA-Levofloxacin interactions had functional relevance in the context of cellular metabolism. Levofloxacin interacts with a G4 motif to compromise translation of the Y-box binding protein 1 (YBX1) mRNA, possibly contributing to the side effects observed with Levofloxacin consumption. Off-target binding of Levofloxacin to the $5^{\prime }$ -UTR of YBX1 mRNA alters the structural dynamics of certain regions in the RNA, potentially modulating the loading of translation initiation factors (Fang et al., 2023).

Connecting mechanisms of side-effect to an intended effect is a known hypothesis generation strategy in the repurposing literature (Recino et al., 2025). The direct conclusion implies potential repurposing of Levofloxacin to target pathogens with G4 motifs that are pivotal to their life cycles. However, taken together, these instances of molecules approved by SM and FDA interacting with RNP targets require repurposing versus $5^{\prime }$ -UTRs as a more general strategy to combat viral pathogens.

## 4 Virtually screening many potential targets within a $5^{\prime }$ -UTR

A key obstacle to discovering SMs that inhibit viral RNA metabolism is the limited availability of high-resolution structures of intact RNP complexes that orchestrate viral gene expression. We hypothesize that AF3 can generate accurate models of fragments of these RNP assemblies that recapitulate the salient features of the binding interface. Restricting AF3 predictions to the minimal RNP structural fragments necessary to recapitulate the local binding interface simplifies the problem, and therefore will likely improve the quality of such predictions. These AF3-generated models can then be refined using enhanced sampling MD simulations to produce ensembles that explore local binding geometries suitable for virtual screening. In this framework, AF3 prediction functions as a source of reasonable starting coordinates that appropriately constrains the binding interface. This strategy rests on the assumption that for SM virtual screening, the local stereochemical environment primarily dictates docking accuracy and efficacy.

This hypothesis is motivated by the well-established observation that many RBPs recognize short, often degenerate sequence motifs situated within unpaired or conformationally flexible regions of RNA secondary structure (Corley et al., 2020). Currently, the Protein Data Bank PDB contains thousands of distinct RNP complexes, of which a large fraction represent individual domains bound to short stretches of single stranded RNA (Krüger et al., 2018). These structures offer a rich training set from which AF3 can learn fundamental determinants of interfacial protein-RNA recognition. Although distal structural interactions contribute to the overall binding affinity and global features of molecular recognition, they are generally less critical for identifying SM modulators that act at a specific RNP interface.

### 4.1 Case study: the workflow applied to EV-A71’s interaction with hnRNP A1

Taking EV-A71 as a model system, the $5^{\prime }$ -UTR is predicted to fold into six individual stem-loops, all of which have multiple interacting protein partners that contribute to the overall regulation of the viral lifecycle. As discussed in Section 2.1, the first stem-loop (SLI) is critical to viral replication (Lin et al., 2009; Huang and Shih, 2014; Davila-Calderon et al., 2024). The remaining stem-loops (SLII-SLVI) collectively make up the IRES and control viral protein production by modulating its interactions with various protein partners (Thompson and Sarnow, 2003; Tolbert et al., 2017; Davila-Calderon et al., 2020; Davila-Calderon et al., 2024). In its entirety, the $5^{\prime }$ -UTR binds more than 20 proteins that fall into various protein classes such as heterogeneous nuclear ribonucleoprotein (hnRNPs), enzymes, and transcription factors (Abedeera et al., 2024; Lin et al., 2015; Yuan et al., 2018). Thus, by targeting specific RNP complexes, downstream aspects of the viral life cycle that are related to a given RNP complex can be altered.

**FIGURE 2:**
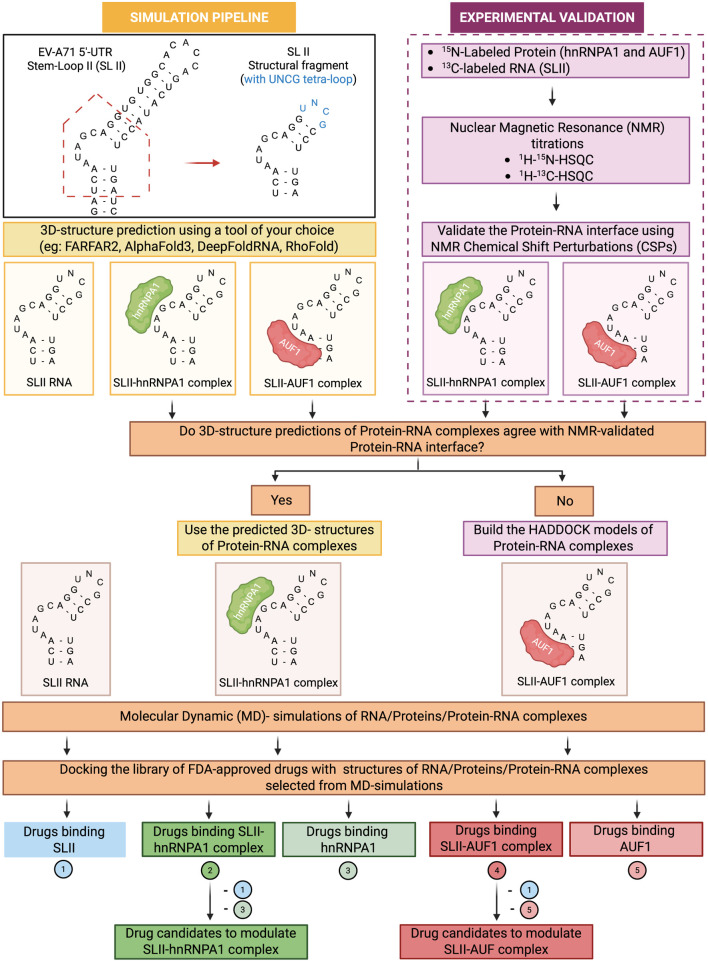
Proposed workflow for identifying SMs that modulate regulatory RNP interfaces. Disease-relevant RNA structural elements and their protein binding partners within $5^{\prime }$ -UTRs of viral or cellular mRNAs are identified through literature, database, or experiment. RNA motifs (e.g., bulge loops) are embedded into minimal structural fragments to recapitulate the local RNP interface and validated using NMR spectroscopy for site-specific RNP interaction mapping. The validated models are subjected to MD simulations to generate conformational ensembles. Pocket detection and ensemble virtual screening using FDA approved compounds prioritize candidate SMs, which are subsequently evaluated with biophysical and functional assays.

Figure 2 illustrates this strategy using the EV-A71 SLII domain and two of its cognate RBPs. The bulge loop of SLII serves as the primary recognition element for both hnRNP A1 and AUF1, which bind competitively to this site to modulate IRES-dependent translation efficiency. Several crystal and NMR structures exist (PDB codes 4YOE, 2LYV, 8X0N, 6DCL, 1 X0F, and 1WTB) for each protein bound to short RNA or DNA fragments resembling the SLII bulge motif ( $5^{\prime }$ -AAUAGCA- $3^{\prime }$ ), underscoring the suitability of this region for structure-guided modeling.

An additional advantage of this minimal fragment approach is that isotopically labeled protein and RNA constructs can be prepared in parallel, enabling NMR chemical shift mapping to directly validate the local RNP binding interface. As outlined in Figure 2, data-driven docking provides a complementary route for generating initial coordinates of the targeted RNP when AF3 predictions are inconsistent with experimentally derived NMR chemical shift constraints (Honorato et al., 2021; 2024).

Our approach to generating an AF3 model of the hnRNP A1-SLII complex suitable for virtual screening involves designing a truncated SLII construct that retains the bulge loop and three base pairs flanking it on each side (SLII-sf). Because the apical loop is not the target of interest, we propose replacing it with a GNRA or UNCG tetraloop, both of which are extensively represented in the PDB and provide stable, well-defined structural caps that are not known to bind hnRNPs. For hnRNP A1, the tandem RRM domains constitute the RNA-binding specificity module; therefore, the N-terminal tandem RRM fragment (UP1) will be paired with the SLII-sf as the input for AF3-guided complex modeling. This targeted approach increases the likelihood that the predicted complex captures the native binding mode, as most RNA binding proteins recognize sequence-specific motifs presented within single-stranded regions of structured RNAs (Hermann and Patel, 2000; Castello et al., 2012; Van Nostrand et al., 2020). By isolating the relevant RNA element as a modular bulge fragment, we focus on the interaction surface where the RBP engages the target through its canonical B-sheet interface. Matched isotopically-labeled protein and RNA constructs should be prepared in parallel, facilitating NMR based validation of the predicted binding interface.

To perform a virtual screen, we first subject the RNP complexes to MD simulations to identify transient and potentially binding pockets for SM modulators. Although MD is a medium-low throughput technique, it provides a physically grounded means of assessing the plausibility of AF3 models; consistency of key intermolecular contacts across the initial model and simulated ensemble lends credibility to both (Hennig, 2025; Krokidis et al., 2025; Perkins-Jechow et al., 2025). The use of adaptive sampling or other enhanced sampling, such as Gaussian-accelerated MD, enables efficient exploration of the conformational landscape and yields an approximate view of the ensemble on a timescale of several weeks using commonly available computational resources (Doerr et al., 2016; Wang et al., 2021). Even shorter timescale simulations can be informative, as cryptic pockets can emerge within hundreds of nanoseconds across replicate trajectories (Meller et al., 2023). Potential binding pockets can be identified using established tools such as ligsite or f-pocket can be used, as well as the RNA specific updates proposed for larger structured RNAs (Veenbaas et al., 2025; Hendlich et al., 1997; Le Guilloux et al., 2009).

**FIGURE 3:**
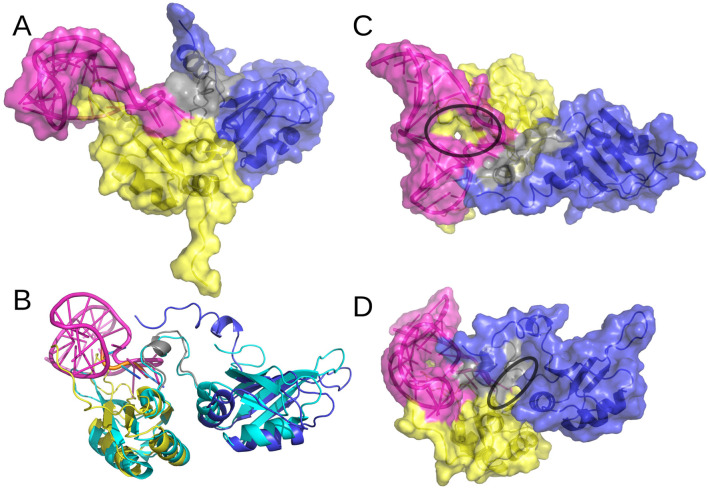
Case study: Fragment design and simulated conformations for the SLII bulge from EV-A71, in complex with the UP1 domain of hnRNPA1. Panel **(A)** shows the AF3 model we used to initialize our simulations. Here, the full-length RNA is in magenta, RRM1 of the simulated conformation is in yellow, the interdomain linker is in grey, and RRM2 is in blue. Panel B shows a relaxed structure from these simulations with PDB 4YOE as a reference. 4YOE is in cyan, with its RNA backbone shown in orange and bases in cyan, with the simulated conformation colored as in Panel **(A)**. Panels **(C,D)** show potentially useful pockets revealed in simulation; here they are highlighted by black ovals. C shows a pocket on the 90-degree rotation downward of the conformation from **(B)**. Panel **(D)** is a novel pocket that emerges between the interdomain linker and the body of RRM2 in some of the states observed in simulation.

Notably, initial simulations have found RNP complexes that are interesting, and starting ensemble cluster-centers that are plausible, when this workflow outlined in Figure 2 is applied to EV-A71 SLII-fr in complex with UP1 (see Figure 3). Docking would then be performed against ensemble conformations that exhibit well-defined pockets using molecules from the Broad drug repurposing hub database, and aggregating results with PopShift (Corsello et al., 2017; Smith et al., 2024).

Ensemble-based virtual screening is particularly important for RNA containing complexes, since RNA conformational dynamics often give rise to pockets that are transient (Chu et al., 2019). Even proteins traditionally considered rigid exhibit substantial pocket dynamics (Mobley et al., 2017; Amaro et al., 2018). PopShift addresses challenges associated with ensemble docking by using a discretized representation, typically a Markov State Model (MSM), to organize and reduce the number of structures required for docking while preserving conformational heterogeneity (Smith et al., 2024). Because PopShift scores the receptor conformations that are preferred by different ligands, it can be used to understand rudimentary allosteric preferences sampled in the ligand-free ensemble, per the conformational selection hypothesis (Changeux and Edelstein, 2011). This could be particularly helpful for class 5 targets (see Figure 1).

### 4.2 Validation, benchmarking, and limitation

The framework outlined here is designed to prioritize functionally relevant RNP interfaces - the regulatory “active sites” of RNA - in virtual screening rather than to predict binding affinities or nominate clinical candidates directly. In this context, ensemble-based virtual screening functions as a triage and hypothesis generation step that identifies candidate SMs capable of engaging structurally defined RNP interfaces. Docking into RNA and RNP remains challenged by conformational heterogeneity, electrostatic complexity, and limitations in current scoring functions, all of which can elevate false-positive rates. Ensemble docking helps mitigate these issues by capturing transient pocket formation that emerges through conformational selection across structural ensembles. Benchmarking strategies such as enrichment analysis, decoy sets, or retrospective recovery of known ligands would further strengthen the framework but fall beyond the scope of this Perspective.

Importantly, the computational framework outlined here must ultimately be validated through orthogonal experimental approaches. As depicted in Figure 2, the proposed workflow incorporates NMR-based validation (Wüthrich, 1986; Clore and Gronenborn, 1989) early in the discovery process, enabling iterative model refinement and decision making in near real time. Selective isotopic labeling of both components of the RNP complex allows simultaneous acquisition of interaction data for each binding partner (Gossert and Jahnke, 2016). Analysis of concentration-dependent chemical shift perturbations provides site-specific insight into intermolecular contacts, and enables precise localization of the amino acid residues and nucleotides that collectively define the binding interface (Williamson, 2013). While NMR spectroscopy is highlighted for its ability to provide site-specific, bi-directional information on RNA and protein interfaces, it represents only one component of the validation toolkit. Depending on system constraints, complementary approaches such as electrophoretic mobility shift assays (EMSAs), isothermal titration calorimetry (ITC), surface plasmon resonance (SPR), footprinting may also be incorporated. The essential requirement is an orthogonal experimental readout demonstrating that the predicted interfaces and ligand interactions correspond to measurable perturbations of the targeted RNP interface.

## 5 Discussion and conclusion

The difficulty of targeting RNA with SMs is often attributed to the absence of well-defined binding pockets comparable to catalytic active sites that guide conventional protein-based drug discovery. However, most functional RNAs exert their regulatory effects through RNA-protein assemblies rather than through RNA structure in isolation. In this context, RNP interfaces can be viewed as the functional equivalents of the “active sites” for RNA-mediated regulation, where specific combinations of RNA structure and protein recognition generate stereochemically discrete surfaces. Structured elements within viral and cellular $5^{\prime }$ -UTRs are particularly well suited to this view, as they organize multiprotein complexes that control gene expression. By focusing on these RNP interfaces, it becomes possible to apply principles of structure-guided SM discovery to RNA-centered regulatory systems and to rationally prioritize SMs capable of biasing RNP equilibria.

Here, we propose that viral RNP targets for therapeutic intervention can be identified more efficiently by integrating AF3 predictions with rigorous biophysical characterization. The core innovation of the workflow is its focus on predicting minimal RNP structural fragments that accurately recapitulate the native binding interface, thereby constraining the search space to the most relevant stereochemical features. By coupling these fragments to adaptive-sampling MD simulations, a broader fraction of the conformational landscape can be sampled. Virtual docking can then be performed on representative cluster centers, enabling an ensemble-based view of ligandability rather than relying on a single static structure. In parallel, NMR chemical shift perturbations provide a rapid and sensitive experimental readout to validate interface fidelity and confirm ligand-induced perturbations in the local RNA environment.

This integrated strategy has the potential to substantially shorten the time required to identify small molecules capable of modulating RNP interactions. Because functional specificity is likely to arise not from a single lock-and-key interaction between small molecules and RNA, but from shifting the equilibria of broader RNP networks, we anticipate that this minimal fragment, ensemble-guided approach will generalize well beyond the systems discussed here. Looking forward, such workflows could form the basis of a new discovery paradigm - one in which predictive modeling, conformational sampling, and targeted biophysics operate synergistically to illuminate previously inaccessible RNP interfaces. As structural prediction engines and biophysical methods continue to mature, this strategy is poised to accelerate the development of therapeutics that modulate RNP assemblies across diverse disease contexts.

In this view, RNP interfaces represent the functional regulatory “active sites” of RNA-centered biological processes, and their systematic identification and interrogation may open new avenues for SM discovery across viral and cellular regulatory networks.

## Data Availability

The original contributions presented in the study are included in the article/supplementary material, further inquiries can be directed to the corresponding author.
